# Assessment of MicroRNA (miR)-365 Effects on Oral Squamous Carcinoma Cell Line Phenotypes

**DOI:** 10.3390/biom11060874

**Published:** 2021-06-12

**Authors:** Jeffrey Coon, Karl Kingsley

**Affiliations:** 1 Department of Clinical Sciences, School of Dental Medicine, University of Nevada-Las Vegas 1001 Shadow Lane, Las Vegas, NV 89106, USA; coonj1@unlv.nevada.edu; 2 Department of Biomedical Sciences and Director of Student Research, School of Dental Medicine, University of Nevada-Las Vegas 1001 Shadow Lane, Las Vegas, NV 89106, USA

**Keywords:** microRNA, miR-365, oral cancer

## Abstract

miR-365 is a microRNA that regulates transcription and has been demonstrated to promote oncogenesis and metastasis in some cancers while suppressing these effects in others. Virtually no information is known about the presence or function of miR-365 in oral cancers. Based upon this information, the primary goal of this project was to evaluate the expression of miR-365 in existing oral cancer cell lines. Five commercially available oral cancer cell lines (SCC4, SCC9, SCC15, SCC25, and CAL27) were obtained and cultured. RNA was then screened by PCR using primers specific for miR-365, as well as matrix metalloproteinase (MMP-2) and a downstream cancer stem cell regulator (NKX2.1), and structural and metabolic standards (beta actin, GAPDH). miR-365 was detected among these oral cancers, and some cells also expressed NKX2.1 and MMP-2, which correlated with miR-365 levels. The relative expression of miR-365, NKX2.1, and MMP-2 RNA was higher than expected. Transfection of miR-365 resulted in a significant increase in proliferation, which was not observed in the negative controls. These data appear to confirm miR-365 expression in oral cancers, which may also be correlated with MMP-2 and NKX2.1 expression. Moreover, the fastest growing oral cancers with the highest viability produced the most miR-365. In addition, miR-365 transfected into cells significantly increased growth, even in normal cells. More research is needed to elucidate the pathways responsible for these observations.

## 1. Introduction

miR-365 is a microRNA that regulates transcription and has been demonstrated to promote oncogenesis and metastasis in some cancers while suppressing these effects in others [1,2]. Studies have shown that miR-365 inhibited breast, liver, and ovarian cancer phenotypes through a variety of interactions with differing targets, such as ADAM10, Bcl-2, and Wnt5a [3,4,5,6,7,8]. However, in other studies, miR-365 has promoted skin and lung cancers through interactions with BAX, Slit2, and ROBO1 [9,10,11].

There is emerging evidence that miR-365 may also function to modulate some oral squamous cell carcinomas via downregulation and interactions with other microRNAs, such as NEAT1/RGS20 and cyclin D1 [12,13]. One study found miR-365 interactions with the transcription factor KRT16, which mediated oral squamous cell carcinoma signal transduction through the beta5/c-met integrin signaling pathway [14]. Other mechanisms involved with miR-365 expression include interactions with NKX2.1 in non-small-cell lung cancers and MMPs in other tissues [15,16,17].

Additional studies have demonstrated that miR-365 interactions may, in fact, be so integral to pancreatic oncogenesis and proliferation that has been identified from expression arrays as one of the select few microRNAs considered to be of significant prognostic value as a circulating biomarker in these cancers [18,19]. Moreover, evidence has suggested that expression and subsequent detection of miR-365 in biofluids may be a critical biomarker in determining whether a pancreatic tumor may be operable [20]. In addition, new studies have revealed that miR-365 (along with miR-135) may play the central and most critical roles in regulating altered functional pathways in triple-negative breast cancers [21,22].

However, due to the variability in miR-365 expression among various cancers and the differential mechanisms of transcriptional regulation observed, these interactions have yet to be fully elucidated in oral tissues and cancers. Due to the lack of conclusive evidence regarding the potential role of miR-365 in oral squamous cell carcinomas, more detailed and specific studies are needed to determine the expression of miR-365 and phenotypic effects in oral cancers [23,24]. Based upon the lack of conclusive information regarding these effects, the primary objective of the current study was to evaluate the expression of miR-365 among available and well-characterized oral squamous cell carcinomas cell lines and to determine any functional relationship with cellular phenotypes.

## 2. Materials and Methods

### 2.1. Experimental Cell Lines

The commercially available oral squamous cell carcinoma lines used in this study were obtained from American Tissue Culture Collection (ATCC; Manassas, VA, USA). Each cell line was verified and cross-checked against the International Cell Line Authentication Committee (ICLAC) database to ensure these were not among the currently known cross-contaminated or misidentified cell lines. Short tandem repeat (STR) profiling results for these six cell lines were compared using the eight STR loci from the ATCC database for cell line authentication.

All cell cultures were maintained in a humidified biosafety level-2 tissue culture chamber at 37 °C. All cultures were maintained in the appropriate media (Dulbecco’s modified Eagle’s medium (DMEM)) with the addition of 10% fetal bovine serum (FBS) and 1% penicillin–streptomycin and 5% CO_2_. Each cell line was passaged 1:3, and doubling time was measured as the time to reach 75–85% confluence, which was the manufacturer’s recommendation (ATCC; Manassas, VA, USA) for maintaining cells in the exponential growth phase.

### 2.2. RNA Extraction

RNA was isolated from each cell line (at baseline in triplicate, *n* = 6) using the Invitrogen TRIzol organic RNA isolation reagent kit (Invitrogen; Carlsbad, CA, USA) and the protocol recommended by the manufacturer. In brief, cell cultures were lysed using 1 mL of RNA isolation reagent (phenol-based) and transferred to microcentrifuge tubes. Then, 0.2 mL of chloroform was added prior to incubation at room temperature for five minutes. Centrifugation was completed at 12,000× *g* of relative centrifugal force (RCF) at 40 °C for 15 min. The RNA-containing upper phase was transferred to a sterile microcentrifuge tube with the addition of 0.5 mL of isopropanol to precipitate the RNA. Following mixing and incubation for an additional five minutes, the RNA was precipitated by centrifugation at 12,000× *g* for five minutes. The supernatant was removed, and the RNA pellet was washed with 75% ethanol prior to another round of centrifugation. Finally, the supernatant was removed, and the RNA pellet was resuspended using 100 uL of sterile distilled water.

### 2.3. RNA Quantification

The isolated RNA was analyzed using the NanoDrop 2000 spectrophotometer from ThermoFisher (Fair Lawn, NJ, USA) and absorbance readings were recorded at A260 nm and A280 nm. The concentration of RNA was determined using absorbance at A260 × the dilution factor and the extinction coefficient (Beer–Lambert law). The absorbance ratio of A260:A280 was used to determine RNA purity. A260:A280 ratios greater than 1.70 can be considered acceptable for use in polymerase chain reaction (PCR) screening assays.

### 2.4. PCR Expression

Expression of RNA was screened using reverse transcription(RT)-PCR and was accomplished using the following primers, synthesized by SeqWright and ThermoFisher Scientific (Fair Lawn, NJ, USA):

Beta actin forward, 5′-GTGGGGTCCTGTGGTGTG-3′; 18 nt, 67% GC, Tm: 69 °C
Beta actin reverse, 5′-GAAGGGGACAGGCAGTGA-3′,18 nt, 61% GC, Tm: 67 °C

Glyceraldehyde 3-phosphate dehydrogenase (GAPDH)
GAPDH forward, 5′-ATCTTCCAGGAGCGAGATCC-3′; 20 nt, 55% GC, Tm: 66 °C
GAPDH reverse, 5′-ACCACTGACACGTTGGCAGT-3′; 20 nt, 55% GC, Tm: 70 °C

miR-365 forward, 5′-ATAGGATCCTGAGGTCCCTTTCGTG-3′; 25 nt, 52% GC, Tm: 70 °C
miR-365 reverse, 5′-GCGAAGCTTAAAAACAGCGGAAGAGTTTGG-3′; 30 nt, 47% GC, Tm: 72 °C

NKX2.1 forward, 5′-CAGGACACCATGAGGAACAGCG-3′; 22 nt, 59% GC, Tm: 71 °C
NKX2.1 reverse, 5′-GCCATGTTCTTGCTCACGTCCC-3′; 22 nt, 59% GC, Tm: 71 °C

Matrix metalloproteinase (MMP-2)
MMP-2 forward, 5′-CTTTGCAGGAGACAAGTTCTGG-3′; 22 nt, 50% GC, Tm: 66 °C
MMP-2 reverse, 5′-TTAAGGTGGTGCAGGTATCTGG-3′; 22 nt, 50% GC, Tm: 66 °C

RT-PCR screening was performed using total RNA and the ABgene Reverse-IT One-Step RT-PCR Kit and a mastercycler gradient thermocycler from Eppendorf (Hamburg, Germany). One ug of RNA was used for each reaction. Reverse transcription was performed for 30 min at 47 °C, followed by denaturation. Thirty cycles of PCR were then performed, using the standard program involving denaturation, annealing at the appropriate primer temperature for each primer pair, and five minutes of extension at 72 °C. PCR reaction products were visualized using 4% agarose gels and ethidium–bromide using a Kodak Gel Logic 100 Imaging system and 1D Image Analysis Software from Eastman Kodak (Rochester, NY, USA). PCR band densitometry and relative mRNA expression levels were quantified using Adobe Photoshop—Image Analysis tools.

### 2.5. Transfection and Proliferation Assays

miR-365 cDNA was generated using the aforementioned primer sets. RNA for miR-365 was generated using RNA polymerase and RT-PCR-generated cDNA. Transfection of the cell lines was accomplished using the Stratagene mammalian transfection kit (Calcium phosphate CaPO4). In this process, cells were incubated with the CaPO_4_ reagent, and RNA produced from PCR-generated cDNA following DNase treatment [10 ug] was added for approximately three hours. Cells were subsequently plated in 96-well tissue culture assay plates at a standard concentration of 1.2 × 10^5^ cells/mL and were allowed to proliferate for one, two, and three days. Cells were fixed at each time point with 10% buffered formalin and subsequently stained with Crystal violet. Growth was measured for transfected (experimental) and non-transfected (negative control) cells using a BioTek Elx808 microplate reader (BioTek Instruments; Winooski, VT, USA) using absorbance at 595 nm.

### 2.6. Statistical Analysis

Differences in measured parameters between transfected and non-transfected cells were measured using a continuous variable (absorbance). Appropriate parametric statistical analysis was performed on measured differences between continuous variables. Two-tailed Student’s *t*-tests were used to determine any statistically significant measurement differences between experimental and control cells using an alpha level of 0.05.

## 3. Results

**Figure 1:**
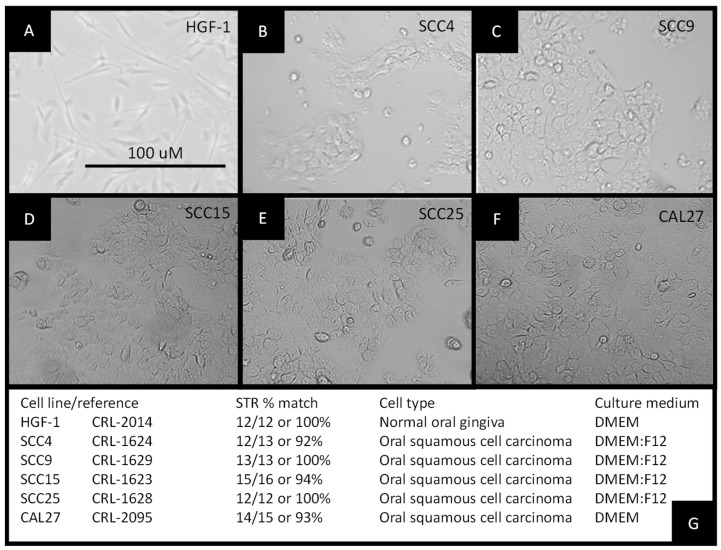
Establishment of experimental cell cultures: morphology of (**A**) normal, non-cancerous human gingival fibroblast HGF-1, (**B**) squamous cell carcinoma SCC4, (**C**) squamous cell carcinoma SCC9, (**D**) squamous cell carcinoma SCC15, (**E**) squamous cell carcinoma SCC25, and (**F**) squamous cell carcinoma CAL27 cells; (**G**) STR profiling was used to confirm cellular identification.

All cell lines were cultured according to the test manufacturer’s recommendations and protocols (Figure 1). All cells were cultured for a minimum of 10 passages prior to RNA extraction. No contamination or other experimental confounders were noted. The slowest growing cells were the normal, non-cancerous human gingival fibroblasts, HGF-1 (Figure 1A). The oral squamous cell carcinomas were found to proliferate either more slowly proliferation identified as SCC4 and SCC9, with doubling times of 5–7 days (Figure 1B,C), or more rapidly growing identified as SCC15, SCC25, and CAL27, with doubling times of 2–3 days (Figure 1D–F). Cell authentication was also performed using short tandem repeat (STR) profiling to confirm cell line identification (Figure 1G).

**Figure 2:**
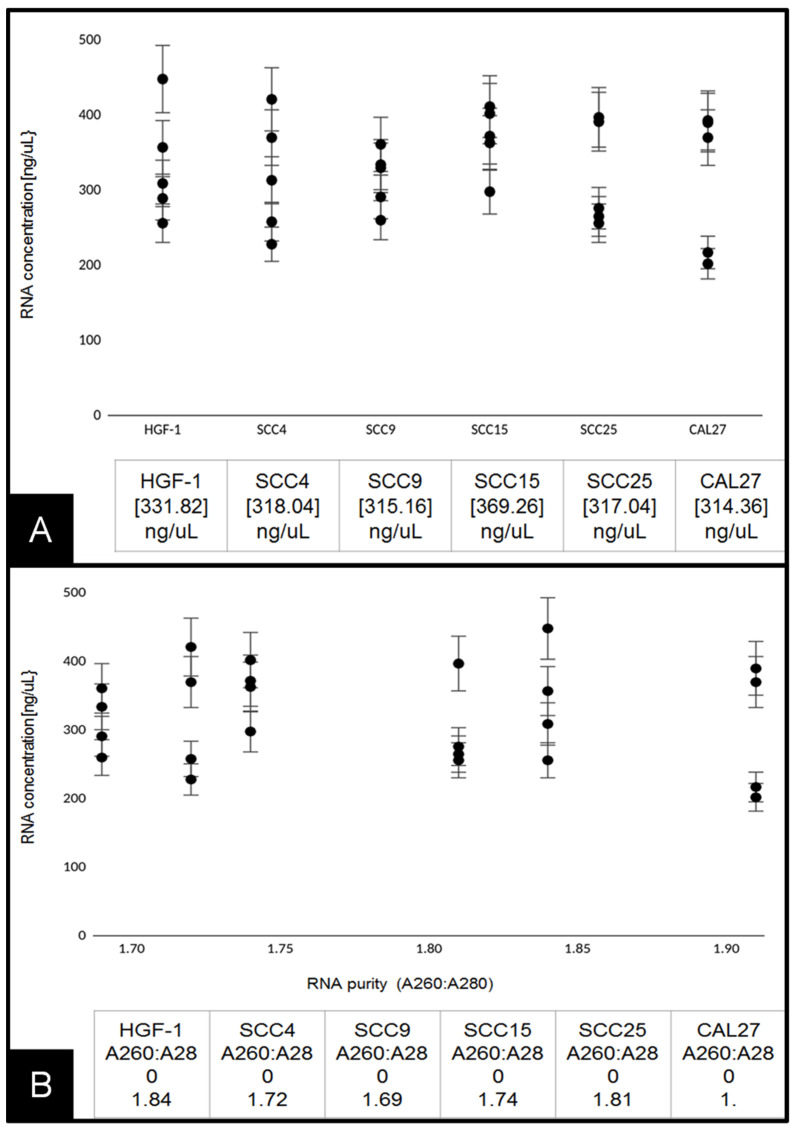
Analysis of extracted RNA: (**A**) concentrations of total RNA extracted from live-cell cultures were similar among HGF-1, SCC4, SCC9, SCC15, SCC25, and CAL27 cells ranging from 314 to 369 ng/uL, *p* = 0.2939; (**B**) average RNA purity (A260:A280 ratio) plotted against RNA concentration. Average absorbance ratio measured at A260 nm and A280 nm revealed ranges between 1.69 and 1.91, with no significant difference between cell lines or discernable associations with overall concentration, *p* = 0.784.

RNA extraction and analysis of experimental cell lines were performed at approximately 70% confluence (Figure 2A). These data demonstrated that the average RNA concentration from each cell line (*n* = 5 per cell line) was similar, ranging from 314.36 ng/uL to 369.26 ng/uL, *p* = 0.2939. The results of each RNA isolation were well within the manufacturer’s range of 100–1000 ng/uL and similar to those obtained from other studies [25]. To determine whether RNA purity was associated with any differences in experimental outcomes, the absorbance ratio of A260:A280 was plotted against concentration (Figure 2B). These data demonstrated that the A260:A280 ratio ranged between 1.69 and 1.91, with no discernable association with RNA concentration, *p* = 0.784.

**Figure 3:**
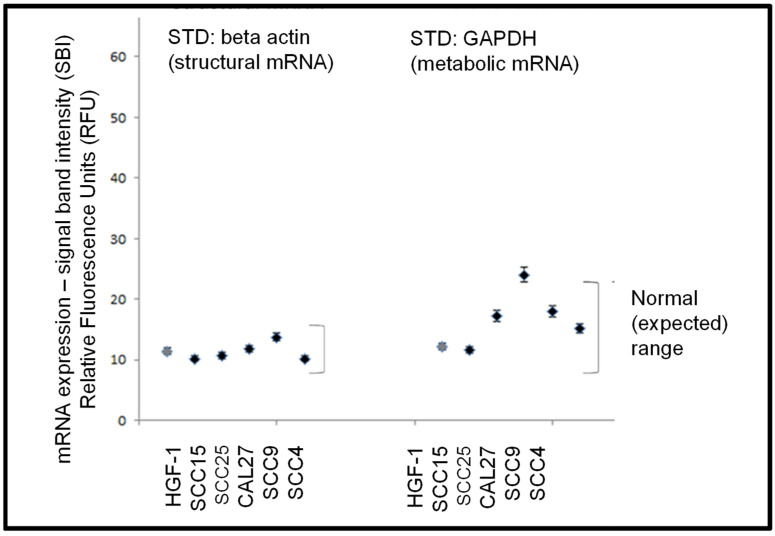
Expression of standard biomarker mRNA. RT-PCR screening of total RNA from each cell line demonstrated mRNA expression levels of the cytoskeletal biomarker beta actin and the metabolic biomarker GAPDH was demonstrated within a defined (expected) range for these cell lines.

Following the confirmation of sufficient RNA concentration and quality, RT-PCR screening was performed to determine the expression of standard biomarkers (Figure 3). These data demonstrated that levels of mRNA for the structural cytoskeleton biomarker beta actin were detectable in all cell lines examined within a fairly narrow (expected) range. In addition, mRNA for the metabolic biomarker GAPDH enzyme in the glycolytic pathway was detected in all cell lines within a broader but well-defined (expected) range.

**Figure 4:**
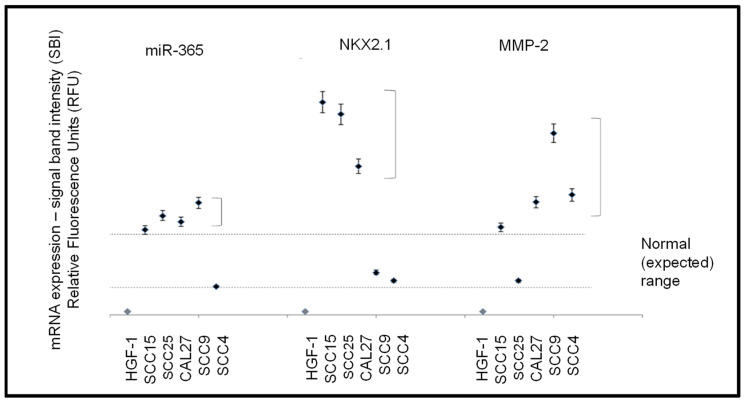
Expression of mRNA and microRNA. The normal, non-cancerous cell line HGF-1 did not express miR-365, NKX2.1, or MMP-2 with variable expression among the oral squamous cell carcinoma lines. Higher than expected levels of miR-365 were detected in all SCC lines except SCC4, *p* = 0.044. Higher than expected mRNA expression levels of NKX2.1 were observed in all SCC lines except SCC4 and SCC9, *p* = 0.038. MMP-2 was expressed in high levels among all SCC cell lines, except SCC25, *p* = 0.041.

After experimental confirmation of structural and functional biomarkers (beta actin, GAPDH), RNA from each cell line was screened for expression of miR-365, and mRNA for NKX2.1 and MMP-2 (Figure 4). These data revealed that the normal, non-cancerous cell line HGF-1 did not exhibit detectable expression of miR-365, NKX2.1, or MMP. However, variable expression was observed among the oral squamous cell carcinoma lines. For example, miR-365 was highly expressed (above the ranges exhibited by the structural and metabolic biomarkers) in SCC15, SCC25, CAL27, and SCC9, *p* = 0.044, but not in the slowest growing SCC4 cell line.

More variable expression was observed with NKX2.1, with higher-than-expected mRNA levels among the three fastest-growing SCC cell lines (SCC15, SCC25, and CAL27, *p* = 0.038) and detectable but lower levels in SCC9 and SCC4 cells. MMP-2 expression was also highly expressed in most of the SCC cell lines (SCC15, SCC9, SCC4, and CAL27, *p* = 0.041), with lower but detectable expression in SCC25 cells.

**Figure 5:**
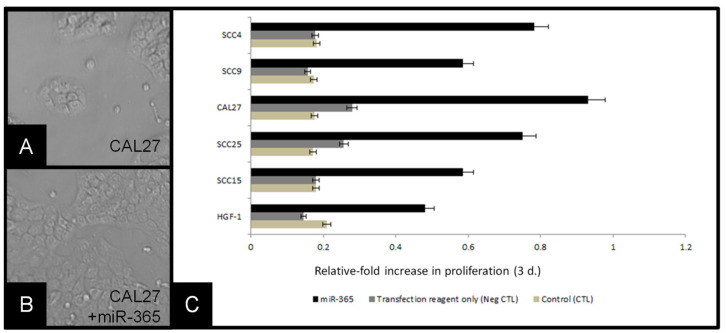
Transfection of cells with miR-365 induced proliferation: (**A**) CAL27 cells in culture, (**B**) CAL27 cells transfected with miR-365, (**C**) molecular confirmation of miR-365 RNA generation, (**D**) relative-fold change in growth of cell lines, compared with baseline (control) and transfection reagent alone (negative control) revealed miR-365 experimental transfection of cell-induced robust changes to proliferation over three days, *p* = 0.014.

To evaluate the effects of miR-365 expression in oral squamous cell carcinomas more thoroughly, transfections were used to increase cellular RNA levels (Figure 5). Following RT-PCR generation of cDNA, RNA polymerase was used to generate large amounts of miR-365 for calcium phosphate transfections into existing cell lines, including CAL27 cells (Figure 5A). Three-day assays revealed strong increases in growth among the miR-365 transfected cells, such as CAL27 (Figure 5B). Quantification of these data demonstrated that miR-365 induced significant changes in growth among all cell lines evaluated (including the normal, non-cancerous control line HGF-1), which were not observed in the presence of transfection reagent alone (control), *p* = 0.014.

## 4. Discussion

Due to the variation in miR-365 expression among various types of cancers and the differential types of phenotypic effects associated with this expression, the primary objective of this study was to provide a more detailed and extensive elucidation of the effects of miR-365 expression among oral squamous cell carcinoma cell lines. Although three previous studies have found miR-365 expression among CAL27 or SCC25 cells, this is the first study to demonstrate that SCC4, SCC9, and SCC15 also express miR-365 [12,13,14]. This may suggest that miR-365 is widely expressed among many types of oral cancers—although more research will be needed for a definitive determination. In addition, this study is also the first to demonstrate concomitant expression of NKX2.1 among these oral cancers, a key signaling mediator in both lung and cervical cancers that is directly regulated by the miR-365 expression [8,26].

In addition, previous studies have found associations between miR-365 expression and MMP-9 regulation [17,27]. However, the results of this study suggest that MMP-2 may also be highly upregulated in oral squamous cell carcinomas expressing both NKX2.1 and miR-365, which confirm previous observations of the importance and potential role of MMP-2 upregulation in oral carcinogenesis and pathogenesis, although more research is needed to determine if any mechanisms for direct or indirect modulation can be established [28,29,30].

Understanding of the role of miR-365 in oncogenesis and proliferation appears to be evolving as more studies confirm the utility of this microRNA as a diagnostic biomarker for breast and liver cancers [31,32,33]. Evidence has emerged that miR-365 modulates and mediates transcriptional activity within many types of cancers, and may also be exported in extracellular vesicles or exosomes that can provide diagnostic information as well as providing prognostic value for evaluating and treating clinical patients [34,35]. Future studies might investigate the potential for oral cancers to secrete miR-365 within exosomes with the potential to improve both diagnostic and prognostic capabilities using non-invasive methods of detection from saliva or other biofluids [36,37,38,39].

Moreover, evidence that a select set of specific microRNAs may be “master” modulators of gene expression, signaling pathway control, and chemotherapy resistance in oral tumorigenesis, development, and progression makes the discovery and elucidation of any individual microRNA with any of these potential abilities, such as miR-365, of significant importance [40,41]. As the most recent evidence now suggests, extracellular vesicles containing specific microRNA cargo may also have the ability to modulate specific phenotypes, including angiogenesis in nearby lymph nodes and lymph tissues, as well as the localized immune response to the primary tumor and associated metastases [42,43]. Recent evidence from this group has demonstrated the export of miR-365 from oral squamous cell carcinomas into extracellular vesicles and exosomes; these results, which highlight the potential for exogenous miR-365 to modulate cellular phenotypes, become more relevant with potential clinical and prognostic implications [39,44].

## 5. Conclusions

These data appear to confirm miR-365 expression in oral squamous cell carcinomas, which may also be correlated with MMP-2 and NKX2.1 expression. Moreover, the fastest growing oral squamous cell carcinoma cell lines produced the most miR-365, and miR-365 transfected into cells was sufficient to increase growth significantly, even in normal cells. Given that extracellular vesicles and exosomes containing microRNAs have been demonstrated to modulate angiogenesis and localized immune responses, more research is needed to elucidate whether these effects can also be observed clinically in oral cancers. Moreover, as evidence emerges that miR-365 can be exported by oral cancer cell lines into extracellular vesicles or exosomes, these observations must be confirmed in vivo, which may be useful to improve diagnostic and therapeutic potential among oral cancer patients.

## Data Availability

Data are available upon request.

## References

[1] Zhan B., Lu D., Luo P., Wang B. (2016). Prognostic Value of Expression of MicroRNAs in Non-Small Cell Lung Cancer: A Systematic Review and Meta-Analysis. Clin. Lab..

[2] Ebrahimi S., Hosseini M., Ghasemi F., Shahidsales S., Maftouh M., Akbarzade H., Parizadeh S.A., Hassanian S.M., Avan A. (2016). Circulating microRNAs as Potential Diagnostic, Prognostic and Therapeutic Targets in Pancreatic Cancer. Curr. Pharm. Des..

[3] Liu F., Zhuang L., Wu R., Li D. (2019). miR-365 inhibits cell invasion and migration of triple negative breast cancer through ADAM10. J. Balk. Union Oncol..

[4] Zhang J., Zhang Z., Wang Q., Xing X.J., Zhao Y. (2016). Overexpression of microRNA-365 inhibits breast cancer cell growth and chemo-resistance through GALNT4. Eur. Rev. Med. Pharmacol. Sci..

[5] Gao J., Zhao P., Chen X., Wang W., Li Y., Xi W., Zhang W., Hu P., Wang T., Shan L. (2016). miR-365 inhibits proliferation and promotes apoptosis of SOSP9607 osteosarcoma cells. Xi Bao Yu Fen Zi Mian Yi Xue Za Zhi.

[6] Zhu Y., Zhao H., Rao M., Xu S. (2017). MicroRNA-365 inhibits proliferation, migration and invasion of glioma by targeting PIK3R3. Oncol. Rep..

[7] Li M., Yang Y., Kuang Y., Gan X., Zeng W., Liu Y., Guan H. (2017). miR-365 induces hepatocellular carcinoma cell apoptosis through targeting Bcl-2. Exp. Ther. Med..

[8] Wang Y., Xu C., Wang Y., Zhang X. (2017). MicroRNA-365 inhibits ovarian cancer progression by targeting Wnt5a. Am. J. Cancer Res..

[9] Zhou M., Zhou L., Zheng L., Guo L., Wang Y., Liu H., Ou C., Ding Z. (2014). miR-365 promotes cutaneous squamous cell carcinoma (CSCC) through targeting nuclear factor I/B (NFIB). PLoS ONE.

[10] Zhou L., Gao R., Wang Y., Zhou M., Ding Z. (2017). Loss of BAX by miR-365 Promotes Cutaneous Squamous Cell Carcinoma Progression by Suppressing Apoptosis. Int. J. Mol. Sci..

[11] Wang Y., Zhang S., Bao H., Mu S., Zhang B., Ma H., Ma S. (2018). MicroRNA-365 promotes lung carcinogenesis by downregulating the USP33/SLIT2/ROBO1 signalling pathway. Cancer Cell Int..

[12] Huang G., He X., Wei X.L. (2018). lncRNA NEAT1 promotes cell proliferation and invasion by regulating miR-365/RGS20 in oral squamous cell carcinoma. Oncol. Rep..

[13] Liu X., Shang W., Zheng F. (2018). Long non-coding RNA NEAT1 promotes migration and invasion of oral squamous cell carcinoma cells by sponging microRNA-365. Exp. Ther. Med..

[14] Huang W.C., Jang T.H., Tung S.L., Yen T.C., Chan S.H., Wang L.H. (2019). A novel miR-365-3p/EHF/keratin 16 axis promotes oral squamous cell carcinoma metastasis, cancer stemness and drug resistance via enhancing β5-integrin/c-met signaling pathway. J. Exp. Clin. Cancer Res..

[15] Moisés J., Navarro A., Santasusagna S., Viñolas N., Molins L., Ramirez J., Osorio J., Saco A., Castellano J.J., Muñoz C. (2017). NKX2-1 expression as a prognostic marker in early-stage non-small-cell lung cancer. BMC Pulm. Med..

[16] Kang S.M., Lee H.J., Cho J.Y. (2013). MicroRNA-365 regulates NKX2-1, a key mediator of lung cancer. Cancer Lett..

[17] Li G., Bu J., Zhu Y., Xiao X., Liang Z., Zhang R. (2015). Curcumin improves bone microarchitecture in glucocorticoid-induced secondary osteoporosis mice through the activation of microRNA-365 via regulating MMP-9. Int. J. Clin. Exp. Pathol..

[18] Johansen J.S., Calatayud D., Albieri V., Schultz N.A., Dehlendorff C., Werner J., Jensen B.V., Pfeiffer P., Bojesen S.E., Giese N. (2016). The potential diagnostic value of serum microRNA signature in patients with pancreatic cancer. Int. J. Cancer..

[19] Tan X., Zhou L., Wang H., Yang Y., Sun Y., Wang Z., Zhang X., Gao F., Li H. (2018). Differential expression profiles of microRNAs in highly and weakly invasive/metastatic pancreatic cancer cells. Oncol. Lett..

[20] Yan Q., Hu D., Li M., Chen Y., Wu X., Ye Q., Wang Z., He L., Zhu J. (2020). The Serum MicroRNA Signatures for Pancreatic Cancer Detection and Operability Evaluation. Front. Bioeng. Biotechnol..

[21] Bertoli G., Cava C., Corsi F., Piccotti F., Martelli C., Ottobrini L., Vaira V., Castiglioni I. (2021). Triple negative aggressive phenotype controlled by miR-135b and miR-365: New theranostics candidates. Sci. Rep..

[22] Ding L., Gu H., Xiong X., Ao H., Cao J., Lin W., Yu M., Lin J., Cui Q. (2019). MicroRNAs Involved in Carcinogenesis, Prognosis, Therapeutic Resistance and Applications in Human Triple-Negative Breast Cancer. Cells.

[23] Rupaimoole R., Slack F.J. (2017). MicroRNA therapeutics: Towards a new era for the management of cancer and other diseases. Nat. Rev. Drug Discov..

[24] Lin S., Gregory R.I. (2015). MicroRNA biogenesis pathways in cancer. Nat. Rev. Cancer.

[25] Moody M., Le O., Rickert M., Manuele J., Chang S., Robinson G., Hajibandeh J., Silvaroli J., Keiserman M.A., Bergman C.J. (2012). Folic acid supplementation increases survival and modulates high risk HPV-induced phenotypes in oral squamous cell carcinoma cells and correlates with p53 mRNA transcriptional down-regulation. Cancer Cell Int..

[26] Chen P.M., Cheng Y.W., Wang Y.C., Wu T.C., Chen C.Y., Lee H. (2014). Up-regulation of FOXM1 by E6 oncoprotein through the MZF1/NKX2-1 axis is required for human papillomavirus-associated tumorigenesis. Neoplasia.

[27] Zimta A.A., Cenariu D., Irimie A., Magdo L., Nabavi S.M., Atanasov A.G., Berindan-Neagoe I. (2019). The Role of Nrf2 Activity in Cancer Development and Progression. Cancers (Basel).

[28] Li Y., Wang Y., Sun H., Zhang Y., Li H., Cong X., Yin W., Song W. (2018). Association Between Matrix Metalloproteinase-1, 2, 3 Polymorphisms and Oral Cancer Risk: A Meta-Analysis. Genet. Test. Mol. Biomarkers.

[29] Boelen G.J., Boute L., d’Hoop J., EzEldeen M., Lambrichts I., Opdenakker G. (2019). Matrix metalloproteinases and inhibitors in dentistry. Clin. Oral Investig..

[30] Deng W., Peng W., Wang T., Chen J., Zhu S. (2019). Overexpression of MMPs Functions as a Prognostic Biomarker for Oral Cancer Patients: A Systematic Review and Meta-analysis. Oral Health Prev Dent..

[31] Han J.G., Jiang Y.D., Zhang C.H., Yang Y.M., Pang D., Song Y.N., Zhang G.Q. (2017). A novel panel of serum miR-21/miR-155/miR-365 as a potential diagnostic biomarker for breast cancer. Ann. Surg. Treat. Res..

[32] He R.Q., Pang Y.Y., Zhang R., Liang H.W., Li C.Y., Ma J., Feng Z.B., Peng Z.G., Chen G. (2017). Down-regulation of MiR-365 as a novel indicator to assess the progression and metastasis of hepatocellular carcinoma. Int. J. Clin. Exp. Pathol..

[33] McDonald A.C., Vira M., Shen J., Sanda M., Raman J.D., Liao J., Patil D., Taioli E. (2018). Circulating microRNAs in plasma as potential biomarkers for the early detection of prostate cancer. Prostate.

[34] Binenbaum Y., Fridman E., Yaari Z., Milman N., Schroeder A., Ben David G., Shlomi T., Gil Z. (2018). Transfer of miRNA in Macrophage-Derived Exosomes Induces Drug Resistance in Pancreatic Adenocarcinoma. Cancer Res..

[35] Min Q.H., Wang X.Z., Zhang J., Chen Q.G., Li S.Q., Liu X.Q., Li J., Liu J., Yang W.M., Jiang Y.H. (2018). Exosomes derived from imatinib-resistant chronic myeloid leukemia cells mediate a horizontal transfer of drug-resistant trait by delivering miR-365. Exp. Cell Res..

[36] He C., Zheng S., Luo Y., Wang B. (2018). Exosome Theranostics: Biology and Translational Medicine. Theranostics.

[37] Ludwig N., Whiteside T.L., Reichert T.E. (2019). Challenges in Exosome Isolation and Analysis in Health and Disease. Int. J. Mol. Sci..

[38] Khodashenas S., Khalili S., Forouzandeh Moghadam M. (2019). A cell ELISA based method for exosome detection in diagnostic and therapeutic applications. Biotechnol. Lett..

[39] Coon J., Kingsley K., Howard K.M. (2020). miR-365 (microRNA): Potential Biomarker in Oral Squamous Cell Carcinoma Exosomes and Extracellular Vesicles. Int. J. Mol. Sci..

[40] Rishabh K., Khadilkar S., Kumar A., Kalra I., Kumar A.P., Kunnumakkara A.B. (2021). MicroRNAs as Modulators of Oral Tumorigenesis-A Focused Review. Int. J. Mol. Sci..

[41] Geretto M., Pulliero A., Rosano C., Zhabayeva D., Bersimbaev R., Izzotti A. (2017). Resistance to cancer chemotherapeutic drugs is determined by pivotal microRNA regulators. Am. J. Cancer Res..

[42] Castilho R.M., Squarize C.H., Almeida L.O. (2017). Epigenetic Modifications and Head and Neck Cancer: Implications for Tumor Progression and Resistance to Therapy. Int. J. Mol. Sci..

[43] Yap T., Pruthi N., Seers C., Belobrov S., McCullough M., Celentano A. (2020). Extracellular Vesicles in Oral Squamous Cell Carcinoma and Oral Potentially Malignant Disorders: A Systematic Review. Int. J. Mol. Sci..

[44] Barlak N., Capik O., Sanli F., Karatas O.F. (2020). The roles of microRNAs in the stemness of oral cancer cells. Oral Oncol..

